# Magnetic Resonance Microscopy for Assessment of Morphological Changes in Hydrating Hydroxypropylmethyl Cellulose Matrix Tablets *In Situ*

**DOI:** 10.1007/s11095-012-0837-y

**Published:** 2012-08-25

**Authors:** Piotr Kulinowski, Anna Młynarczyk, Przemysław Dorożyński, Krzysztof Jasiński, Marco L. H. Gruwel, Bogusław Tomanek, Władysław P. Węglarz

**Affiliations:** 1 Department of Magnetic Resonance Imaging, Institute of Nuclear Physics PAN, ul. Radzikowskiego 152, 31-342 Kraków, Poland; 2 Department of Pharmaceutical Technology and Biopharmaceutics Pharmaceutical Faculty, Jagiellonian University, ul. Medyczna 9, 30-688 Kraków, Poland; 3 National Research Council Canada, Institute for Biodiagnostics, 435 Ellice Avenue, Winnipeg, Manitoba Canada R3B 1Y6

**Keywords:** controlled release, hydroxypropylmethyl cellulose (HPMC), pharmaceutical magnetic resonance imaging (MRI), swellable matrix tablets, T_2_ magnetic resonance relaxometry

## Abstract

**Purpose:**

To resolve contradictions found in morphology of hydrating hydroxypropylmethyl cellulose (HPMC) matrix as studied using Magnetic Resonance Imaging (MRI) techniques. Until now, two approaches were used in the literature: either two or three regions that differ in physicochemical properties were identified.

**Methods:**

Multiparametric, spatially and temporally resolved T_2_ MR relaxometry *in situ* was applied to study the hydration progress in HPMC matrix tablets using a 11.7 T MRI system. Two spin-echo based pulse sequences—one of them designed to specifically study short T_2_ signals—were used.

**Results:**

Two components in the T_2_ decay envelope were estimated and spatial distributions of their parameters, *i.e.* amplitudes and T_2_ values, were obtained. Based on the data, five different regions and their temporal evolution were identified: dry glassy, hydrated solid like, two interface layers and gel layer. The regions were found to be separated by four evolving fronts identified as penetration, full hydration, total gelification and apparent erosion.

**Conclusions:**

The MRI results showed morphological details of the hydrating HPMC matrices matching compound theoretical models. The proposed method will allow for adequate evaluation of controlled release polymeric matrix systems loaded with drug substances of different solubility.

## INTRODUCTION

### Structural Characteristics and Analysis of Matrix Systems

Hydrophilic matrix systems are often used for controlled release (CR) of drugs. The controlled release of an active pharmaceutical ingredient is obtained by the formation of a rubbery layer on the surface of the matrix. This layer acts as a barrier for inward liquid penetration and provides a controlled release of the dissolved drug. Although the working principle of such a system is simple, the exact spatial and temporal behavior of matrix during the drug dissolution is a complex phenomenon which includes the interplay of a number of physico-chemical processes.

Hydroxypropylmethyl cellulose (HPMC) is one of the best-known excipients used for the preparation of a CR polymeric matrix systems (1). The well-defined structure, the wide range of polymer types and the consistency of the samples make HPMC the polymer of choice for CR systems.

In order to identify the morphology of CR systems, the evolution of matrix swelling was studied with photography, video, and light scattering techniques (2–5). In other studies Attenuated Total Reflection Fourier Transform Infrared (ATR-FTIR) microscopy (6), also combined with macro photography (7), was used for the identification of the water/polymer/drug distribution within the matrix system. Various methods based on ultrasound techniques and penetrometric textural analysis have also been proposed to study the mechanical properties of the hydrated part of CR systems (8,9).

The concept of moving fronts is often applied to describe the structure of swelling polymeric matrix systems (5). It is derived from optical photography studies of the behavior of polymer-drug systems during hydration. Colombo *et al.* (2) introduced two fundamental boundaries (moving fronts): the glass transition boundary where glassy material is transformed into a gel matrix, and the erosion boundary where the matrix completely disappears by dissolution or erosion (2). The third zone, called the drug diffusion boundary, was identified in between these two fronts. In this area drug dissolution and diffusive transport occur. On the other hand, Gao and Meury (4) defined three similar boundaries: (1) the true penetration boundary where water transport takes place through the pores of the glassy carrier; (2) a phase transition boundary where the glassy material is transformed into a gel matrix and (3) an erosion boundary (dissolution front) where the matrix completely disappears by dissolution or erosion. Despite these extensive studies, information on the properties and nature of these particular areas is still limited. This is mainly due to fact that most of the optical or chemical methods cannot provide simultaneous information on both spatial distribution of water in the matrix and its physical properties. Supplementary techniques *e.g*. penetrometry, applied for the identification of polymeric matrix strength are destructive and cannot be applied together with noninvasive recording of the CR system behavior (9).

Magnetic Resonance Imaging (MRI) is a method ideally suited for the visualization of water ingress into the polymer matrix and is widely used for controlled release applications (10,11). In most of the MRI studies so far, the gel layer and core (unhydrated part of the matrix) were distinguishable (12–17). For example, in the work by Tajarobi *et al.* (16) two fronts (swelling and erosion) were identified in tablets containing pure HPMC, HPMC + mannitol (freely soluble substance) and HPMC + dicalcium phosphate (insoluble substance).

Some authors present a three-region approach (18–22). Kojima *et al.* identified an interface layer between the gel and dry glassy core (18). In our previous work we have reported on the observation of three regions in the swelling HPMC matrices (19). Consequently, a dry core region, a hydrogel formation region and a hydrogel region were identified (20). In subsequent work the interface region was assigned to the swollen glassy polymer (21).

### Spatially Resolved MR Relaxometry

Relaxation times T_1_ (spin–lattice) and T_2_ (spin-spin) associated with molecular energy exchange can be used for probing mobility of molecules in the investigated objects (23,24). T_2_ values lower than ∼50 μs occur in solids (low molecular mobility), values of ∼1 ms in plastic (or swollen) phases and values of tens of milliseconds to seconds in liquids (high molecular mobility). Dependence of T_1_ on molecular mobility is more complicated. Generally, it is long in solids and in liquids, the minimum (short) T_1_ value is reached in the region of intermediate molecular mobility. In addition T_1_, and to a lesser extend T_2_, depend on the strength of the magnetic field used for the MR experiments.

Spatially resolved T_2_ relaxometry allows parametric images in terms of amplitudes (corresponding to the proton density (PD) of the detected pool of protons) to be obtained and the relaxation constants (T_2_) of the decay envelope to be extracted (24). Such parametric images (comprising T_2_ and PD values) allow to distinguish phases of different water/matrix mobility within the hydrated HPMC material. Early studies were performed using a single spin-echo technique repeated several times with a different echo time (12,13,25). Then the Multi Slice – Multi Echo (MSME) pulse sequence, based on the Carr-Purcell-Meiboom-Gill (CPMG) principle, became a basic MRI technique for spatially resolved T_2_ imaging (26). MSME has been used by many authors to study relaxation parameters of hydrated HPMC tablets (14–18). The samples were prepared using various methods and studied under different experimental conditions using variable temperatures, solvents, pH, etc. In most cases mono-exponential data fitting (only one T_2_ value) was applied to describe the hydration and consecutive swelling kinetics of HPMC based tablets. As the experimental conditions differ in each study, comparison of results is difficult. Moreover, no results are reported for long and short T_2_ relaxation times measured in a single study.

Probably the first spatially resolved T_2_ studies of HPMC matrices was performed by Rajabi-Siahboomi *et al*. in 1996 and by Fyfe *et al.* in 1997 (13,25). Rajabi-Siahboomi obtained a set of images using a spin-echo sequence with several echo times in the range of 4.5–76.5 ms at 11.7 T, using HPMC tablets hydrated in bi-distilled water at 37°C. Fyfe *et al*. assessed T_2_ relaxation of axial swelling of the tablet at 9.4 T. They acquired several one-dimensional images in an axial direction using a Spin-Echo imaging sequence with varying echo-time from 2 to 128 ms (2, 4, 16, 32, 96, 128, 64, 24 and 3 ms). A single image was acquired in 3 min., while the total acquisition time was relatively long, approximately 30 min. T_2_ profiles as well as related intensity profiles were presented. In 2002, Kojima *et al.* (14) showed 1D profiles of T_2_ obtained at 4.7 T. Echo times were set between 20 and 240 ms at an image slice thickness of 5 mm. While ultra-pure water at room temperature was used as a solvent, T_2_ relaxation times in the external (gel) layer up to ∼1 s were reported. The minimum registered T_2_’s were of the order of hundreds ms. In the following study by Tritt-Goc *et al.*, 64 echoes were collected using the MSME sequence with echo time increments of 10 ms (with the last echo at 640 ms) using a 7 T MR spectrometer (15). An alkaline solvent (pH = 12) was used at 37°C. In this work T_2_ profiles with the corresponding PD profiles, *i.e.* estimated amplitude of CPMG decay, were shown together. Another study was presented by Tajarobi *et al.* (16) and performed at 9.4 T. In this case pure HPMC matrices, as well as matrices loaded with freely soluble and poorly soluble drug substance, were studied. Matrices were investigated in a flow-through cell using a phosphate buffer at 37°C (100 mM, pH 6.8, *I* = 0.1) as solvent. As in the previous study, the MSME pulse sequence was used with the first echo registered at 10 ms and T_2_ decay was sampled up to 200 ms. Swelling and erosion fronts were determined from radial and axial T_2_ profiles. The swelling front was positioned at the lowest assessed T_2_ (40–50 ms) value. The solvent boundary was assumed an erosion boundary (T_2_ of the solvent was in the range of 600–700 ms). Laity *et al.* performed experiments with a multi-echo sequence with a short echo time interval of 2.52 ms, covering up to 40.32 ms with 16 echoes at 7 T (17). The dissolution medium was phosphate-buffered saline (PBS) at pH 7.4 and the swelling study was performed at ambient temperature. In their work the matrix consisted of HPMC K4M and other excipients (micro-crystalline cellulose, lactose). Chen *et al.* used a T_2_ preconditioned Rapid Acquisition with Relaxation Enhancement (RARE) pulse sequence to obtain eight images with echo-times in the range 18.1-2578.1 ms (22). The short acquisition time (2.5 min) was obtained using a low spatial resolution of 0.469 mm.

Spatially resolved T_2_ measurements of hydrated HPMC systems should probe T_2_ decay envelopes to assess relaxation components of tens to hundreds of ms. Some authors sampled T_2_ decay in a long TE regime which resulted in a loss of the short T_2_ data (14–16,22). Others worked with short TE but the echo-train was too short to acquire, and properly analyze, signal with long a T_2_ relaxation time (17). Mikac *et al.* (27) combined two MRI pulse sequences including one dedicated to signals characterized by short T_2_ relaxation times and applied the method to xantan gum matrices. All these previous studies used a mono-exponential decay model for data interpretation. In these studies it was also assumed that the structure of the hydrated matrix consisted of two regions *i.e.* the core and the gel. Considering the above-mentioned disadvantages, we have applied multiparametric MRI approach (spatially resolved T_2_ MR relaxometry) to describe morphology details of hydrating HPMC matrices.

Our work is complementary to the work published recently by Chen *et al.* (22). The work presented by Chen *et al.* was targeted to obtain a high temporal resolution sacrificing spatial resolution (roughly 0.5 mm in plane resolution). Their main interest was the initial period of hydration. Studies with a higher temporal resolution to observe the initial stage of matrix hydration were also performed by van der Weerd and Kazarian (6) and Kazarian and van der Weerd (7) using ATR-FTIR chemical imaging. Concerning structural details, results obtained by Chen *et al.* are similar to the ones previously observed by our group (19–21), mainly due to the comparable echo times used. On the other hand, our approach has roots in two parameter analysis (T_2_ and corresponding proton density) performed by Tritt-Goc *et al.* (15).

In the current study our goal was to obtain a high spatial resolution and to assess morphological details based on the molecular properties of the hydrated matrix reflected in MR detectable parameters (T_1_, T_2_ and proton density).

## MATERIALS AND METHODS

### Materials

HPMC–Metolose 65 SH 400 cP and Metolose 65 SH 10,000 cP (Shin-Etsu Chemical Co., Ltd. Tokyo, Japan) were used for the preparation of the matrix tablets. The composition of the tablets was kept very simple to eliminate the influence of other technological factors on the water penetration into the polymeric matrix. The tablets were made using standard equipment and procedures as applied to research and development studies in pharmaceutical labs. Tablets, 9 mm in diameter and a height of ∼3 mm (200 mg) were prepared from polymers via direct compression using a 10-station ERWEKA TRB 10 press. The mean hardness of the tablets was 6.27 kG, the friability was 0.25–0.42%.

### Experimental Protocol

The hydration of tablets was carried out in 50 mL of 0.1M HCl at room temperature (∼22°C). After the desired hydration time (30, 60, 120 and 180 min) the medium was carefully removed, while protecting the sample against solvent evaporation. The sample was put into a home-built MR probe and a set of MR measurements was performed after which the sample was disposed. To obtain data at the next hydration time a new sample was hydrated independently.

### MRI Data Acquisition

**Fig. 1:**
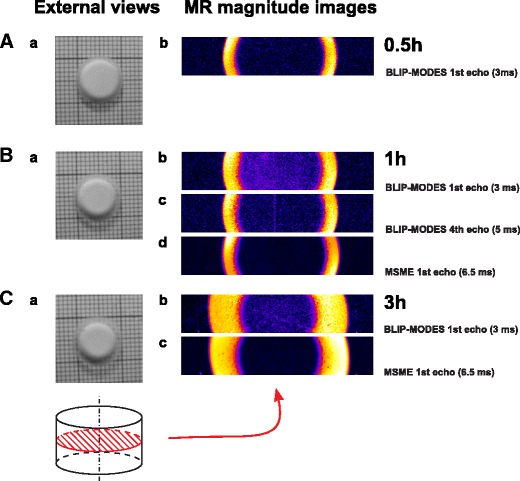
Examples of external views (photo images **A**a, **B**a and **C**a) and the corresponding MR images (**A**b, **B**b-d, **C**b and **C**c) of HPMC 400 cP obtained at different hydration times and different echo times. Schematic view of the tablet is shown at the bottom. External view is top view of the tablet. MR images were performed in the slice marked by red cross-section.

MRI was performed using a 11.7 T (500 MHz) vertical bore magnet (Oxford Instruments U.K.) equipped with a water-cooled, self-shielded gradient system SGRAD 123/72/S with an inside diameter of 72.5 mm and an Avance console (Bruker, Germany) with a ParaVision 3.0.2 operating system. A home-built radio-frequency saddle coil (10 mm ID, 10 mm length) was used. Imaging was performed at room temperature (22°C). 2D MR images of spatial distribution of T_1_ and T_2_ relaxation times were obtained on a single slice in a radial plane (coronal slice) of the hydrating tablet (see schematic view of the tablet in Fig. 1).

#### T_1_ (Spin–Lattice) Relaxation Time Imaging

The first phase of the MRI experiment was to obtain the spatial distribution of T_1_ relaxation times. This parameter provides information on the hydration dynamics of the selected polymers. Spatial distribution of T_1_ relaxation time was obtained using the Fast Imaging with Steady-state Precession (FISP) sequence (26). The imaging sequence parameters were: TE/TR = 2/4 ms, Number of accumulations = 3, Number of images = 40, Matrix size = 128 × 128 × 1, Slice thickness = 1 mm, Field of view = 25 × 25 mm. Inversion time was 400 ms for the first image and then 160 ms apart for the following 39 images.

#### Multiparametric T_2_ (Spin-Spin) Relaxation Time Imaging

The long time scale of the T_2_ decay envelope (up to 208 ms with resolution of 6.5 ms) was probed using the MSME imaging pulse sequence where 6.5 ms was chosen as minimum available echo time. The following parameters were applied: TE/TR = 6.5/4000 ms, number of accumulations = 2, number of images = 32, matrix size = 256 × 256 × 1, slice thickness = 1 mm, Field of view = 15 × 15 mm.

Short time scale of T_2_ decay envelope (up to 12 ms with resolution of 1 ms) was probed using the Broad Line Imaging Package Modes (BLIP_MODES) MRI pulse sequence (ParaVision 3.0, Bruker, Germany). The method is a single slice, single echo routine approach to broad line samples, featuring standard 2D and 3D Fourier imaging, T_1_ and T_2_ imaging (weighting or image series) with emphasis on short TE and on minimum adjustments and modifications upon changing between these modalities. It consists of a hard (nonselective) excitation pulse of recommended 125 microseconds length and a selective (gauss shape) refocusing pulse of 500 microseconds length. The sequence parameters were: TE/TR = 3/500 ms. Number of accumulations = 2, Number of echos = 10, Matrix size = 256 × 256 × 1, Slice thickness = 1 mm, Field of view = 15 × 15 mm. The first image was acquired after 3 ms (minimum available echo time) while the next 9 images were acquired within 1 ms apart.

### Data Analysis

All multi echo magnitude image data sets were analyzed by custom developed MATLAB (The MathWorks, Inc.) scripts. An image mask was created to separate signal containing pixels from background noise and the tablet image was shifted to the image matrix center. Then, nonlinear least-squares curve fitting (MATLAB *lsqnonlin* with Levenberg-Marquardt algorithm) was applied pixel-by-pixel in order to obtain parametric results. In case of MSME data sets, we used two-parameter model function:

1 $$ {S_{{MSME}}}(t) = {S_{{0MSME}}} \cdot \exp \left( { - \frac{t}{{{T_{{2L}}}}}} \right) $$

where: *t* – time, *S*_0*MSME*_ – estimated amplitude of *T*_*2*_ decay envelope at time *t = 0*, *T*_*2L*_ – estimated T_2_ decay constant. For such a two-parameter model function of T_2_ decay envelope, *S*_0*MSME*_ is proportional to the density of mobile protons (PD), contained in each voxel of hydrated polymer matrix.

For BLIP_MODES data set analysis a three-parameter exponential function was chosen:

2 $$ {S_{{BLIP}}}(t) = {A_S} \cdot \exp \left( { - \frac{t}{{{T_{{2S}}}}}} \right) + {A_L} $$

where: *A*_*S*_ – estimated amplitude of *T*_*2*_ decay envelope at *t = 0*, *T*_*2S*_ – estimated T_2_ decay constant, *A*_*L*_ – baseline. By choosing a three-parameter exponential model function we assume that the baseline value (A_L_) will comprise signal of the long T_2_ component.

As a result of the above fitting method, each image pixel was assigned the following values: T_2L_, A_L_, T_2S_ and A_*S*_. It should be emphasized, that amplitudes of both components (A_L_, A_*S*_) were obtained exclusively from BLIP_MODES data. The amplitudes were the main criterion for matrix structure assessment.

The 1D distribution of estimated parameters along the radius of the tablet (radial profiles) were obtained by radial averaging of the fitted parameters over a 60° pie slice. We decided to perform numerical analysis on a 60° pie slice in order to be able to compare tablets of various composition at different hydration time as we could always find such a non distorted piece of tablet. Deformation of the tablet shape occurred especially at longer hydration times of 2–3 h, however it was observed also after one hour of hydration. Slight deviations from circular symmetry were observed even in images which at the first glance appeared to be not deformed.

## RESULTS

Figure 1 presents photo images and the coronal slice of the corresponding MR magnitude images obtained using MSME and BLIP_MODES, for the HPMC 400 cP tablets after 0.5, 1 and 3 h of hydration, respectively. The MR imaging slice is shown as a red cross-section in the schematic view of the tablet at the bottom of the figure. Pictures of the tablets show only an external view of the overall swelling of the polymer, while MR images show evolution of the internal structure of the hydrating tablet. The sets of images were obtained at consecutive echo times (see Fig. 1 for examples) and each pixel signal intensity was fitted as described in the Data Analysis section.

**Fig. 2:**
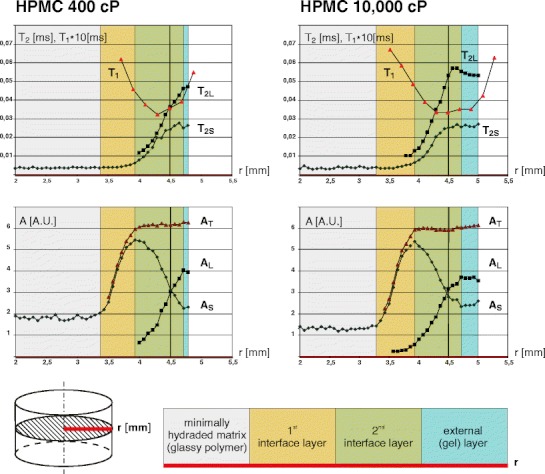
T_2_ values (top row) and signal amplitudes (lower row) of two components in the T_2_ decay envelope together with T_1_ relaxation times (upper row) *vs*. tablet radius at 1 h of hydration for HPMC 400 (left) and 10,000 cP (right) tablets. T_2L_ - T_2_ relaxation time of long component, T_2S_ - T_2_ relaxation time of short component, T_1_ - T_1_ relaxation time, A_S_ - amplitude of short component, A_L_ - amplitude of long component. Color scheme used: gray - minimally hydrated matrix (solid like), yellow - the first interface layer, green - the second interface layer, blue - firm gel layer. Initial (before hydration) tablet’s border (at 4.5 mm) is marked with a solid vertical line.

Spatially resolved T_2_ MR relaxometry provided four parameters for each location (voxel) of the hydrating HPMC polymeric matrix *in situ*: amplitudes (A_L_, A_S_ ) and corresponding T_*2*_’s (T_2L_, T_2S_), describing long and short components respectively. The short component refers to the signal amplitude with the short T_2_ relaxation time while the long component refers to the component of the signal amplitude with the long T_2_ relaxation time. Amplitudes are related to the PD of the two detected pools of protons and the sum of both amplitudes (A_T_ = A_S_ + A_L_) equals the total detected signal (total PD) at a particular spatial location. After radial averaging over a 60° pie, the results can be presented as 1D radial plots of the parameters (*i.e.* radial profiles; see Fig. 2).

Figure 2 shows the spatial distribution of the four parameters along the radius of the hydrated HPMC matrices (marked as a thick red line in the schematic view of the tablet). Complete results, consist of amplitudes and corresponding T_2_ values *vs.* tablet radius (amplitude and T_2_ profiles) are presented in two separate graphs. As an example both polymers (HPMC 400 cP and 10,000 cP) after 1 h of hydration are shown.

The layers in the radial profiles were identified according to the characteristics of the pixel parameter profile behavior (*i.e*. increasing, decreasing or constant). The selection criteria and description of the particular layers are presented below:

1. The first interface layer (marked yellow)
  Criteria of selection: only one signal component exists; its amplitude increases towards the water phase. This component is identified as the short component.
  Detailed description: As seen from Fig. 2, at a certain position looking from the matrix center, the signal amplitude of the short component (A_S_) increases steeply and reaches a maximum. However, the T_2_ value of this component (T_2S_) does not change substantially. Across this layer the amplitude of the short component (A_S_) constitutes practically 100% of the total detected signal (A_T_).
2. The second interface layer (marked green)
  Criteria of selection: two components exist; their parameters change over a wide range but in a systematic manner, across this layer.
  Detailed description: the long component, characterized by A_L_ and T_2L_, appears. Across this layer the amplitude of the long component (A_L_) increases away from the center of the tablet. At the same time the amplitude of the short component (A_S_) decreases. This phenomenon occurs while T_2_ values of both components (T_2L_ and T_2S_) increase. This second interface layer is the most interesting region of the matrix (“amplitude crossing” layer). “Amplitude crossing” occurs approximately when the T_2_ value of the short component (T_2S_) reaches a plateau. The amplitude of both components to the observed signal reach a plateau when the T_2_ value of long component (T_2L_) reaches its plateau.
3. Gel layer (marked blue)
  Criteria of selection: both signal components exist; their parameters do not change substantially as a function of the distance from the center of the tablet.

From the position in the matrix where the total amount of detected solvent (A_T_) reaches a maximum (at a radius of approximately 3.9 mm) it does not change significantly, for increasing radius. However the properties of the system in terms of T_2S_, A_S_, T_2L_ and A_L_ do change until the system reaches the steady state (gel) at the most outer part of the matrix. This is true for first two hydration times (0.5 and 1 h). At 2 and 3 h of imbibition (not shown), A_T_ decrease slightly towards the exterior of the tablet.

T_1_ relaxation time profiles have a different shape than the T_2_ profiles. The T_1_ profile decreases, reaches a minimum and increases towards the tablets boundary. It should be noted that the T_1_ profile was treated as a single exponential phase (for justification/explanation see Discussion section).

**Fig. 3:**
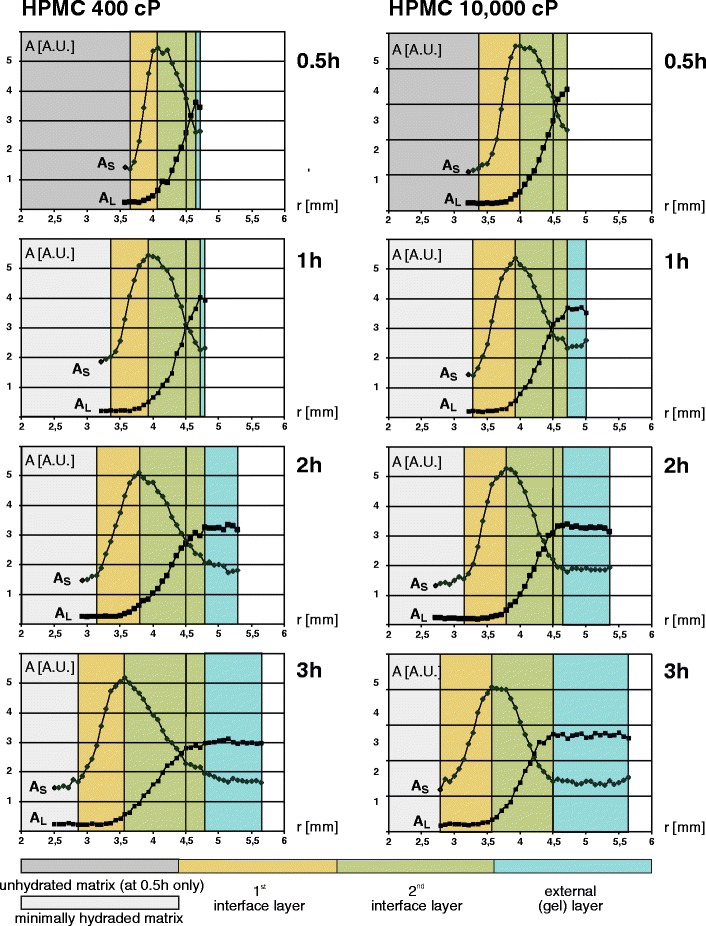
Temporal evolution of the matrices – amplitudes of T_2_ decay envelope components *vs.* tablet radius at 0.5, 1, 2 and 3 h of hydration for HPMC 400 (left) and 10,000 cP (right) tablets. A_S_ - amplitude of the short component, A_L_ - amplitude of the long component. The following layers are marked: dark gray - unhydrated matrix at 0.5 h of hydration only, light gray - minimally hydrated matrix (solid like), yellow - the first interface layer, green - the second interface layer, blue - firm gel layer. Original tablet’s border (at 4.5 mm) is marked with a solid vertical line.

Temporal evolution of the HPMC 400 cP and 10,000 cP systems by the means of amplitude (PD) profiles is shown in Fig. 3 on the left and right hand side, respectively. As in the Fig. 2, the layers in the matrix are marked with colors. Overall swelling of both polymers is similar. But in case of HPMC 10,000 cP, the firm gel layer is thicker at any point in time, while for HPMC 400 cP this layer is thinner at the expense of the second interface layer. For matrices hydrated for 1 h, detectable signal in T_2S_/A_S_ radial profiles exist in the center of the matrix (see Fig. 2). For both types of matrices, the region in the center of tablets is characterized by components having a very short T_2_ (average 3.6 ms for HPMC 400 and 3.8 ms for HPMC 10,000, not shown) and an average signal amplitude of 1.80 ± 0.08 a.u. for HPMC 400 and 1.30 ± 0.07 a.u. in the case of the HPMC 10,000 according to the parametric approach. For comparison, the total amplitudes in the fully hydrated region for both polymers were 5.97 ± 0.59 a.u. and 6.00 ± 0.07 a.u., respectively.

**Fig. 4:**
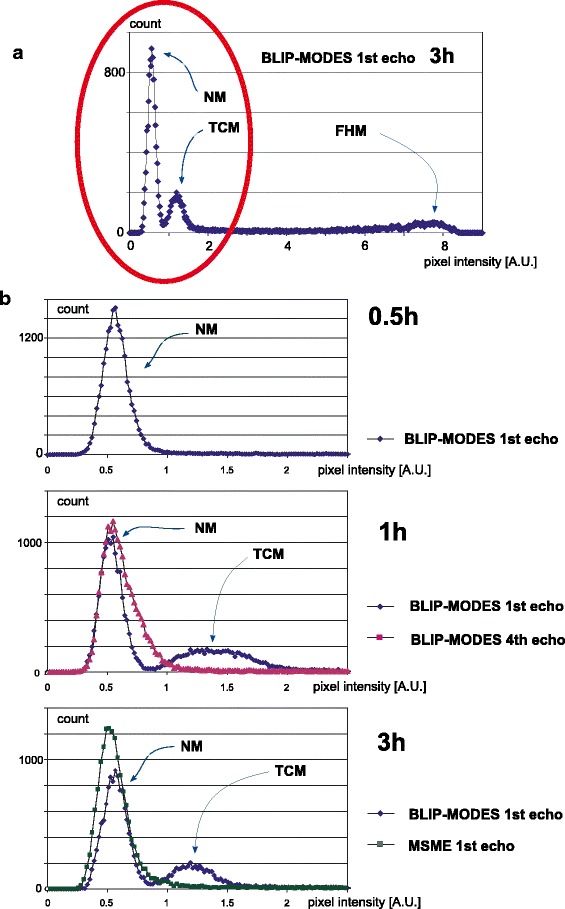
Intensity histograms of the images from Fig. 1. (**a**) Full histogram of image C2 from Fig. 1. (**b**) Histograms restricted to the low intensities only. Histogram modes: NM – noise level, TCM – tablet center signal, FHM – fully hydrated region.

Figures 1 and 4 were prepared to show a signal of very low intensity (distinct from noise) that appears in the center of the tablet. Figure 1 presents magnitude images obtained using BLIP_MODES (at 1st and 4th echo) and MSME (image acquired at the first echo). Prior to the histogram preparation a gaussian blurring filter was applied to images with sigma (radius) of 2. Figure 4a shows a full histogram of Fig. 1Cb. Histograms in Fig 4b show fragments of the image intensity histograms covering low signal intensities only up to 25% of the intensities in the fully hydrated region (the region circled red in Fig. 4a).

At 0.5 h for all systems there is no detectable signal from the inner part of the matrix. The signal in the center of the matrix is at the same intensity level as outside of the matrix (noise level at about 0.6 AU). In Fig. 1Ab, only noise mode (NM) exists in the range of interest (Fig. 4b, first panel).

Starting from 1 h of hydration, the inner part of the matrix is brighter than regions outside of the matrix in the BLIP_MODES 1st echo images (Fig. 1Bb, Cb). A second, separate, mode (CTM) occurs centered at about 1.3 AU in the image intensity histogram (Fig. 4b) of Fig. 1Bb. When looking at a histogram of 4th echo image (Fig. 1Bc), there is no distinct mode related to the center of matrix region, only a slight broadening of the mode at ∼0.6 AU can be observed. This signal could not be presented in the profiles in Fig. 3. Although detectable, this signal suffers from very low signal to noise ratio (SNR) and rapid signal decay. As a result, reliable parametric data could not be obtained for this region. The signal originates from protons of low mobility located in the solid like part of the matrix characterized by T_2_ relaxation times <1 ms. The second mode does not appear in histograms of MSME images, even after 3 h of hydration (Fig. 4b, third panel).

Image analysis results described above were reflected in profiles presented in Figs. 2 and 3. Two different types of matrix were distinguished in the center (core) of the tablet: dark gray region denoted as unhydrated region (at 0.5 h of hydration); light gray region denoted as minimally hydrated region (starting from 1 h of hydration).

## DISCUSSION

Multicomponent T_2_ relaxation is expected in a system of hydrated amorphous polymer with two separate phases (glassy polymer with an intrinsic T_2_ shorter than 50 μs and liquid water with an intrinsic T_2_ of the order of 1 s) before hydration (28). Apparent values of T_2_ and T_1_ during the hydration process are caused by interactions and corresponding magnetization exchange between water molecules and exchangeable protons of the polymer (*e.g.* hydroxyl groups) (15,29–31). In low hydration regime (initial hydration) a short T_2_ component is dominating, however, a gradual increase of the long T_2_ component is observed when moving radially from the tablet center. Presence of multiple T_2_ components is most likely related to the existence of pools of water molecules with a different interaction with the polymer. At low hydration (or close to tablet center) T_2_ is dominated by water strongly coupled to polymer binding sites. For regions with higher hydration, contribution from more mobile water molecules within water clusters dominates. Besides signal from water, contributions from protons contained in polymer chains mobilized in a water environment are likely be present in the signal as well. As T_1_ is about one order of magnitude larger than T_2_, it is not unexpected to detect multiple T_2_ components while only one averaged T_1_ component in such a binary system of a polymer and water (29). Such situations have indeed been observed during HPMC hydration (see Fig. 2). The dependence of T_1_ values on distance from the tablet center (high T_1_ for inner and outer part while reaching minimum for intermediate region) can be explained on the basis of Bloembergen-Purcell-Pound theory relating NMR relaxation times with correlation times of molecular motion, which is slow in solid (glassy) phase and fast in liquid phase (24).

In this study MRI was used to assess the spatial distribution of parameters (T_2_s and corresponding PDs) of two components in the T_2_ decay envelope within the hydrated tablet. Laity *et al.* (17) presented a similar data set, however, the aspect of potential multi-exponential T_2_ decay was not explored. The authors fitted the logarithmic T_2_ decay to linear equation thus they assumed one component. But at least in one case, experimental data obtained from a region with a water content of 40–50%, according to the authors’ calibration, self-evidently does not follow straight line. When relating to the approach introduced in our study, this observation probably relates to the location in the matrix where both (short and long) components had comparable amplitudes.

Based on our results we postulate the existence of the five regions along the radius of the analyzed matrix:

1. unhydrated matrix (no detectable MR signal) located in the inner part of the matrix (only for data acquired at hydration time of 0.5 h);
2. minimally hydrated matrix (solid like) with low mobility protons located in the inner part of the matrix (replacement of unhydrated matrix starting from 1 h of hydration);
3. first interface layer where only the short component exists and towards the outside of the matrix, the amplitude A_S_ increases (high total PD gradient in this region exist);
4. second interface layer where, as a function of distance from the center of the tablet, the amplitude of the short component A_S_ decreases and the long component appears with increasing amplitude (A_L_) (“amplitude crossing” pattern);
5. external (gel) layer, where both components exist with almost constant PD as a function of distance from the center of the tablet.

According to Ueberreiter (32), within the structure of the hydrating polymer one can distinguish pure polymer, an infiltration layer, a solid swollen layer, a gel layer, a liquid layer (diffusion layer as defined by Ju *et al.* (33)) and a pure solvent. In our study, due to the experimental conditions, *i.e.* solvent removal, only the first four layers can be present. The first (inner) interface layer can be attributed to the infiltration layer, and the second (outer) interface layer to the solid swollen region.

Another interpretation of our results can be made with reference to Gao and Meury (4). In their schematic representation of a swelling HPMC matrix structure there are a glassy core, a swollen glassy layer and a gel layer separated by a true water penetration front and a phase transition front. According to this diagram, there is a high water concentration gradient across the swollen glassy layer. Moreover, they distinguish an apparent gel layer and an apparent glassy core with an apparent gel front in between. The apparent gel front appears at a more distant position than the phase transition front, when measured from the center of the tablet. The layer between the fronts is undetectable by optical methods as the region is opaque. We have replaced the phase transition front in this model with the phase transition layer. The following regions (layers) in the hydrating matrix can be distinguished: a dry core, a swollen glassy layer, a phase transition layer, and a gel layer.

A more complex structure of the pure HPMC matrix during hydration was observed by Chirico *et al.* using an optical method (3).The authors observed the presence of a “circular halo” intermediate between the external tablet radius, where the erosion takes place, and the radius at which the water penetrated. The halo was evident in the normalized light intensity profile at 3 days of hydration - it took the form of a “couple of shoulders”. It was also perceptible at 3 hours. Potential region distinction was not explored in their work. The halo was described as an unexpected phenomenon whose origin was not clear. Between halo and core of the matrix a region of lower intensity was also observed. Their analysis of light intensity profiles for tablet gel formation supports the data reported in our work.

Several studies (4,33) assume a continuous increase in solvent concentration across the hydrated polymer matrix (along the tablet radius) even in the gel layer. However our (as well as Tritt-Goc *et al.* (15)) MRI results give an approximately constant water concentration in the hydrated part of the matrix with the exception of a relatively narrow region where a high concentration gradient is observed (for theoretical aspects of liquid transport into and through the polymer matrices see the work by Vesely (34)). Despite the constant solvent concentration, in the inner part of the fully hydrated region, characteristic changes of MR signal amplitudes and T_2_’s of the two components occurs, while the sum of amplitudes (total PD) remains constant. It can be hypothesized, that in this region the water-polymer system undergoes a phase transition from a glassy to a rubbery state. Transition from glassy to rubbery state is accompanied by significant changes in the dynamical state of the polymer molecules as well as its mechanical properties. This happens, when water concentration reaches a certain level. The following factors are essential to form a gel layer: water (plasticizer) concentration, temperature (constant in our experiment) and time. The time factor was pointed out by Kazarian *et al*. (7): they observed deviation from the theoretical value for gel formation, which could be explained by the time needed for gelling. The theoretical value as assessed for equilibrated samples was taken from the study by Hancock and Zografi (35) and was approximately 20% water content. In the external (gel) layer there are no significant changes in T_2_ values of both components as well as in their amplitudes. Coexistence of the two components in a broad spatial range can suggest the non-homogeneous/heterogeneous nature of the hydrated matrix. The only exception to this is the deepest hydrated layer where only the short component exists.

**Fig. 5:**
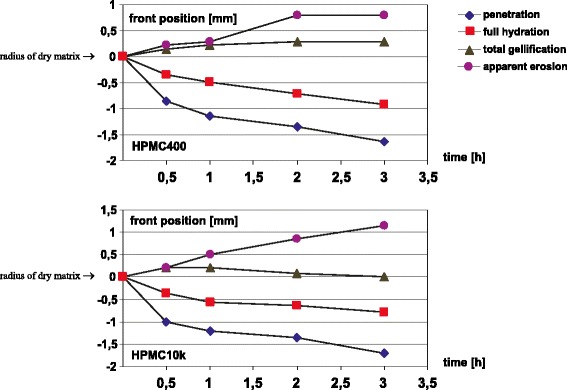
Front temporal movement in radial direction for HPMC 400 cP and HPMC 10,000 cP. The vertical scale 0 denotes the radius of the dry matrix (4.5 mm).

Temporal evolution of the swelling matrix can also be shown in a manner similar to those presented by Tajarobi *et al.* (MRI) (16) or Ferrero *et al.* (optical study) (5) *i.e.* in terms of front evolution. Two sets of front evolution profiles are shown in Fig. 5: for HPMC 400 cP and 10,000 cP. The vertical scale was set in a similar way as for the optical results presented by Ferrero *et al.* (5), where 0 denotes the radius of the dry matrix (in our case 4.5 mm).

Unlike the work by Tajarobi, in our study the amplitude A_S_, A_L_ profiles (PD profiles) were utilized to identify the front’s positions. The deepest front can be denoted as the penetration front. It was set at the position in the matrix where a high solvent gradient across the matrix begins. It marks the boundary between the glassy polymer and the first interface layer. The second front is denoted as the full hydration front. It is positioned where the amplitude of the short component (A_S_) reaches a maximum. It marks the boundary between the first and the second interface layer. Starting from this position, PD in terms of A_T_ does not change substantially across the matrix (PD gradient is almost zero). The third front is denoted as a total gelification front. It marks the boundary between the second interface layer and the gel layer. Starting from this position, the matrix does not undergo a transformation until erosion at the external surface of the matrix. We adopted the term after Kazarian *et al.* (7), however, originally total gelification meant full hydration. We can consider the two fronts detected by Kazarian: a true penetration front and a total gelification front. The front denoted as total gelification appears at a water concentration of about 0.6 w/w. In their work there is a high water concentration gradient in the vicinity of the front. In our data we observed that the total signal amplitude A_T_ (total PD) increases steeply (high concentration gradient) across the 0.5 mm thick layer and then it does not change significantly anymore. We attribute the total gelification, as defined by Kazarian, to the beginning of gelification *i.e.* beginning of the phase transition zone. External front was denoted as apparent erosion front due to absence of solvent. It marks external border of consistent gel which remains after solvent removal. Liquid layer as defined by Ueberreiter (32) can not be observed.

Our results presented in the manuscript can also be compared with those shown in the experimental part of article by Kimber *et al.* (36). In the paper by Kimber *et al.*, radial profiles of water and polymer concentrations for hydrated HPMC matrix at 100 min of imbibition were shown. Their results were obtained by a FTIR method described previously by Kazarian *et al.* (7). In our results, the amplitude of the short T_2_ component increases along the tablet radius starting from the center of the matrix (like the water concentration in the work by Kimber *et al.*) and after full hydration it decreases (like polymer concentration in the work by Kimber *et al.*). Total water concentration (total proton density in our case) in both studies is constant behind the full hydration position. The short T_2_ component originates mainly from “bound” water (water coupled or in chemical exchange with polymer chains, which was hypothesized but not detected by Tritt-Goc *et al.* (15)). The long relaxation component can be regarded as originating from the “free water” pool. On one hand, our results are consistent with those shown by Kimber *et al.* and on the other hand, our results supply us with additional information on water molecular mobility. It allowed us to detect regions and fronts as described in our manuscript.

Figure 5 shows that there is no substantial difference in evolution of the penetration and the full hydration fronts between HPMC 400 cP and 10,000 cP. For HPMC 10,000 cP, starting from 1 h, the total gelification front evolves in parallel to the most inner fronts (penetration and full hydration fronts) toward the center of the matrix, while the apparent erosion front moves outwards, in opposite direction. As a result, a thicker, stable gel layer is produced for HPMC 10,000 cP. The most pronounced difference between the systems was the position of the total gelification front. This corresponds with results obtained by Ferrero, where swelling was more pronounced for higher viscosity grades of HPMC matrices (5).

Glassy-rubbery transformation does not implicate immediate gel formation. The matrix properties change from a glassy core region, through a hydrated opaque viscoelastic polymer to a transparent gel-like matrix and finally to a bulk solvent. It seems that from an MRI point of view, using the term “swelling front” (as used by by Tajarobi *et al.* (16)) is inadequate. Probably that is the reason why Kazarian *et al.* (7) do not use the term “swelling front” when observing HPMC matrices using FTIR/VIS imaging techniques - they used terms “true penetration”, “total gelification” and “erosion” fronts. That is the reason why we named fronts as “penetration”, “full hydration”, “total gelification” and “apparent erosion”. These terms better describe observed phenomena. Some interesting hints can be found in the work by Laity *at al.* (17) which combines MRI and X-ray imaging methods. It was observed that particle displacement could also be observed in the dry core. From an MRI point of view they defined only two fronts “penetration” and “gel-water interface”. Moreover, the terms “penetration”, “phase transition” and “erosion” fronts were used by Chen *et al.* (22).

**Fig. 6:**
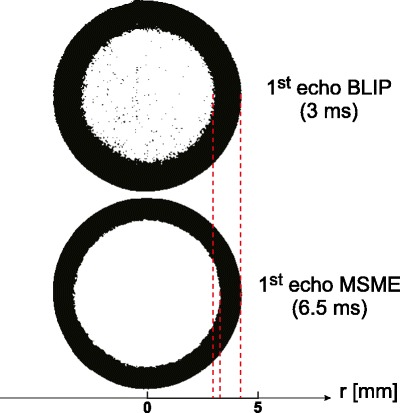
Binary images of total hydrating area at 1 h of hydration in the tablet’s cross-section obtained at first echo using MSME and BLIP_MODES sequences.

The morphology of the hydrated polymer system was the main interest in the current study. Consequently higher priority was given to spatial rather than temporal resolution. In plane spatial resolution in the presented study was 59 × 59 μm at slice thickness of 1 mm, while resolution of previous MRI studies of such systems ranged from 100 to 500 μm. The BLIP_MODES sequence probed the first 12 ms of T_2_ decay with a resolution of 1 ms, while the MSME sequence probed T_2_ decay up to 208 ms with a resolution of 6.5 ms. It has implications concerning MRI detection of the regions possessing different physico-chemical properties. Figure 6 shows binary magnitude images obtained at the first echo for both MR methods used in the study (MSME and BLIP_MODES). Black coloring represents hydrated regions as detected by both methods. It is apparent, that the BLIP_MODES method, with first echo time at 3 ms, detects a larger volume of the hydrated matrix than MSME with a first echo at 6.5 ms.

Echo times ∼2–3 ms were used in early relaxometry studies performed in 90’s by Rajabi-Siahboomi *et al.* and Fyfe (13,25), but in the latest works, most of the authors applied longer echo times in the range 10–20 ms: (14–16,18,22), the only exception being the work by Laity *et al.* (2.5 ms) (17). In these studies only two regions were identified: dry polymer and gel layer. Consequently only two fronts were defined. In our study, we were able to distinguish up to five different regions (and four fronts) in the hydrating matrix.

MRI studies performed in flow-through cells (USP4 and non-pharmacopoeial) which emerged during last few years (see review by Dorożyński *et al.* (11)) usually suffer from spatial resolution and relatively long echo times, but they seem to be useful tool for preformulation studies. Proposed method allows more detailed morphology assessment (existence of more than two regions as detected by MRI methods in hydrating HPMC matrix was confirmed) and can be used as a prerequisite for such studies.

When interpreting MRI results, the impact of echo time on the detection of the matrix hydration, especially at low water content, should be accounted and thoroughly discussed. For example, in our previous work performed using an USP4 apparatus (T_1_/T_2_ weighted magnitude images obtained using flow-insensitive Spin-Echo imaging sequence), histogram based image segmentation into three regions could be performed based on the triple-modal image intensity distribution (21). Subsequently the regions were assigned as a dry glassy core region, an interface layer and a gel layer. Due to the relatively long echo time of 19 ms, we observed a slight underestimation of the interface region in favor of the dry core of the matrix. In this case only fully hydrated part of the matrix could be detected only (*i.e.* the second interface and gel layers as described in the current study).

## CONCLUSIONS

In the present work, a new, multiparametric approach to the investigation of hydrated HPMC matrices by MR microscopy (relaxometry) is presented. Spatially resolved T_2_ MR relaxometry, with in plane resolution of 59 μm, provided four parameters for each spatial location (voxel) of the hydrating HPMC polymeric matrix *in situ*. Moreover, spatially resolved T_1_ relaxometry results were shown and discussed.

Two components in the T_2_ envelope coexist across the fully hydrated layer - they change their amplitudes as well as their T_2_ values as a function of tablet radius, while the sum of the component’s amplitudes (total PD) does not change substantially. It demonstrates heterogeneity of the polymer-drug-water system even in the external (gel) layer, where both components exist, however no substantial spatial changes of their parameters is observed in this region.

The multiparametric T_2_ approach reveals detailed structure of the hydrated HPMC matrix:

- The interface layer between dry polymer and gel can be identified and divided into two sub-layers - existence of two interface sub-layers is predicted by theoretical models.
- Five different regions (*i.e.* a dry glassy, a hydrated solid like, two interface layers and a gel layer *vs.* two or three layers obtained in previous studies) or four moving fronts (penetration, full hydration, total gelification and apparent erosion) were all identified by means of MR micro-imaging methods in the swelling HPMC matrix.

The proposed morphology of the hydrated matrix is closer to compound theoretical models than previous MRI experimental studies. High resolution MR relaxometry as presented in the study, provides new, consistent, insight into the hydrating HMPC based polymeric matrix.

## ABBREVIATIONS

ATR-FTIR: attenuated total reflection fourier transform infrared
BLIP_MODES: broad line imaging package modes
CPMC: Carr-Purcell-Meiboom-Gill
CR: controlled release
FISP: fast imaging with steady-state precession
FTIR: fourier transform infrared
HPMC: hydroxypropylmethyl cellulose
MR: magnetic resonance
MRI: magnetic resonance imaging
MSME: multi slice–multi echo
PD: proton density
RARE: rapid acquisition with relaxation enhancement
TE: echo time
TR: repetition time
USP: United States Pharmacopoeia
VIS: macro-photography
